# A small-scale CRISPR mutant library in rapeseed of commercial cultivar Zhongshuang 11

**DOI:** 10.1093/hr/uhag087

**Published:** 2026-03-05

**Authors:** Can Zeng, Jianjie He, Jianshuo Li, Shipeng Fan, Mingli Wu, Xin Cheng, Yutian Xia, Dongqing Zhang, Xiaoling Dun, Maoteng Li

**Affiliations:** Biological Seed Industry Research Institute, Xianghu Laboratory, Hangzhou, Zhejiang 311231, China; College of Life Science and Technology, Key Laboratory of Molecular Biophysics of the Ministry of Education, Huazhong University of Science and Technology, Wuhan, Hubei 430074, China; College of Life Science and Technology, Key Laboratory of Molecular Biophysics of the Ministry of Education, Huazhong University of Science and Technology, Wuhan, Hubei 430074, China; Department of Biology, Center for Genomics and Systems Biology, New York University, New York, NY 10003, USA; College of Life Science and Technology, Key Laboratory of Molecular Biophysics of the Ministry of Education, Huazhong University of Science and Technology, Wuhan, Hubei 430074, China; College of Life Science and Technology, Key Laboratory of Molecular Biophysics of the Ministry of Education, Huazhong University of Science and Technology, Wuhan, Hubei 430074, China; College of Life Science and Technology, Key Laboratory of Molecular Biophysics of the Ministry of Education, Huazhong University of Science and Technology, Wuhan, Hubei 430074, China; College of Life Science and Technology, Key Laboratory of Molecular Biophysics of the Ministry of Education, Huazhong University of Science and Technology, Wuhan, Hubei 430074, China; College of Life Science and Technology, Key Laboratory of Molecular Biophysics of the Ministry of Education, Huazhong University of Science and Technology, Wuhan, Hubei 430074, China; Biological Seed Industry Research Institute, Xianghu Laboratory, Hangzhou, Zhejiang 311231, China; Oil Crops Research Institute, Chinese Academy of Agricultural Sciences, Wuhan, Hubei 430062, China; Biological Seed Industry Research Institute, Xianghu Laboratory, Hangzhou, Zhejiang 311231, China; College of Life Science and Technology, Key Laboratory of Molecular Biophysics of the Ministry of Education, Huazhong University of Science and Technology, Wuhan, Hubei 430074, China

## Abstract

Rapeseed (*Brassica napus* L.) is one of the most important oil crops worldwide. In our previous work, we generated a high-throughput CRISPR library whereby a knockout collection was established for rapeseed breeding and functional genomics. However, the collection remains small and several promising candidate genes still await functional validation. Here, we report an update of this collection by constructing a small-scale CRISPR mutant library based on the elite commercial cultivar Zhongshuang 11 (ZS11). We first generated 326 independent T_0_ lines using an optimized protocol for ZS11 transformation and regeneration with a high positive rate of 94.2%. Analysis of the editing outcomes revealed a mutagenesis frequency of 68.4%. We then phenotyped this new collection and unraveled possible key genes underlying the variations in seed oil content (SOC) and plant height. Finally, we functionally validated *BnFAB1B* and *BnEDA32*, two candidate genes identified from our knockout collection. The results confirmed that loss of function of *BnFAB1B* significantly increases SOC, indicating its great agronomic potential, whereas knockout of the nuclear-localized *BnEDA32* severely disrupts seed oil accumulation. This study provides a valuable knockout collection of the elite cultivar ZS11 and new genes for creating superior rapeseed germplasm.

## Introduction

As the world’s second-largest oilseed crop, rapeseed (*Brassica napus* L.) contributes ~13.2% to global oil production [1]. To meet the growing edible oil demand, a key objective in rapeseed breeding is to develop elite germplasm with enhanced yield, seed oil content (SOC), and disease resistance. However, improving these complex traits through conventional breeding methods remains inefficient, which are often time-consuming and labor-intensive [2].

Mutant libraries are essential resources for functional genomics and breeding. Traditional methods, such as chemical (e.g., ethyl methanesulfonate, EMS) [3] or physical mutagenesis (e.g., X-rays, gamma rays, and fast neutrons) [4], generate random genome-wide mutations and have been proven valuable for creating larger scale mutant libraries with genetic diversity. For example, Tang *et al*. utilized EMS mutagenesis to develop a large rapeseed mutant population in the elite commercial cultivar Zhongshuang 11 (ZS11), from which high-oil varieties were successfully identified [5]. Besides, ultrasonic seed treatment was successfully used to establish a large-scale mutant collection, from which cadmium (Cd)-tolerant varieties were obtained [6]. However, these methods typically require screening extremely large populations and subsequent laborious forward-genetic approaches like TILLING to identify causal variants [2].

In recent years, the CRISPR-Cas9 (clustered regularly interspaced short palindromic repeats-associated nuclease) system has revolutionized plant genome editing by enabling precise and targeted modifications [7, 8]. This system offers several key advantages for creating mutant libraries, such as high specificity for simultaneous multigene targeting, direct genotype-to-phenotype linkage, and accelerated identification of causal variants compared to random mutagenesis methods. Moreover, its application in high-throughput forward genetics, through genome-scale CRISPR libraries, has been successfully demonstrated in major crops including rice [9, 10], soybean [11], and cotton [12], accelerating gene discovery and trait improvement. For example, Meng *et al*. generated a rice genome-scale CRISPR library comprising 88 541 sgRNAs targeting 34 234 genes, and produced 84 384 independent T_0_ lines. From this population, they rapidly identified causal genes for leaf color and tiller-angle variation, demonstrating the CRISPR library’s value as efficient, high-throughput resources for functional genomics and breeding.

In our previous work, we developed a pooled CRISPR library targeting 10 480 genes with 18 414 sgRNAs in rapeseed [13] and generated 1104 independent T_0_ lines in the cultivar J9709, which is a widely used line in rapeseed transgenic research [14]. Field-grown mutants exhibited diverse morphological and biochemical variations, including altered plant architecture and SOC. Phenotype-to-genotype analysis identified two candidate genes associated with SOC. Specifically, knockout of one *BnFAB1B* homoeolog (mutant ID: 0228) increased SOC, whereas knockout of four *BnEDA32* (mutant ID: 0313) homoeologs significantly reduced it. These preliminary T_0_ findings highlighted the potential of CRISPR libraries for gene discovery in rapeseed, while also underscoring the need for functional validation in advanced generations.

To expand genetic resources and uncover novel genes, we are enlarging our rapeseed mutant collection. In this study, we have generated 326 independent T_0_ lines in the elite cultivar Zhongshuang 11 (ZS11), a widely planted commercial cultivar in China. Furthermore, we functionally validated the two previously identified candidate genes (*BnFAB1B* and *BnEDA32*) associated with SOC and confirmed their key roles in seed oil metabolism. This work provides valuable resources for rapeseed functional genomics and elite germplasm breeding.

## Results

### Mapping the pooled CRISPR library to ZS11 genome

**Figure 1:**
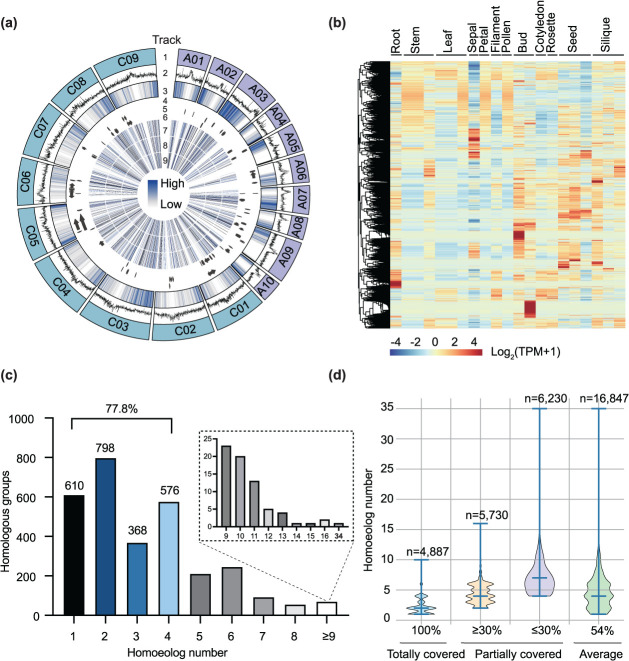
Target analysis of pooled CRISPR library across ZS11 genome. (a) Genomic distribution of target genes. Nineteen chromosomes (Track 1), GC content (Track 2), gene density (Track 3), QTLs for TSW (Track 4), SOC (Track 5) and SL (Track 6), and gene expression levels in seeds at 17 (Track 7), 30 (Track 8), and 42 (Track 9) days after flowering. QTLs, quantitative trait loci; SOC, seed oil content; TSW, thousand seed weight; SL, silique length. (b) Expression profile of target genes. Data are from publicly available RNA-seq datasets of BnIR. TPM, transcripts per million. (c) Homoeologous groups of target genes. (d) Target coverage of sgRNAs.

ZS11 was selected as the parent line to enlarge the rapeseed knockout collection because it is an elite cultivar renowned for its high yield, high SOC, and robust disease resistance [5, 15, 16]. Of the 18 414 sgRNAs in the pooled CRISPR library, 15 426 (83.8%) perfectly aligned to the ZS11 genome with zero mismatches (Fig. S1a) and collectively target 10 078 genes (Tables S1 and S2). These target genes were widely distributed across the genome, with the highest density on chromosome A03, and three subsets colocalized with known quantitative trait loci (QTLs) for SOC, thousand seed weight (TSW), and silique length (SL) (Fig. 1a). Expression profiling further identified 9893 target genes (excluding 185 low-expression genes) across 12 tissues in ZS11 (Fig. 1b), and functional enrichment analysis linked them to multiple key processes such as lipid biosynthesis and fatty acid elongation (Fig. S1b). These results suggested that various abnormal traits might be observed in the resulting knockout collection.

Next, we classified the 10 078 target genes into 3027 homoeologous groups, each corresponding to an *Arabidopsis thaliana* ortholog (Table S3). Most groups (77.8%) contained one to four homoeologs, whereas the largest group comprised 34 homoeologs (Fig. 1c). Through *in silico* CRISPR scanning, we identified 4887 sgRNAs capable of simultaneously targeting all homoeologs within a given group (Fig. 1d). As an example, one sgRNA could target all 10 *BnRPS25E* (*RIBOSOMAL PROTEIN ES25W*) homoeologs at once (Fig. S1c), enabling potential functional dissection of gene redundancy.

### Establishing an efficient protocol for ZS11 transformation and regeneration

**Figure 2:**
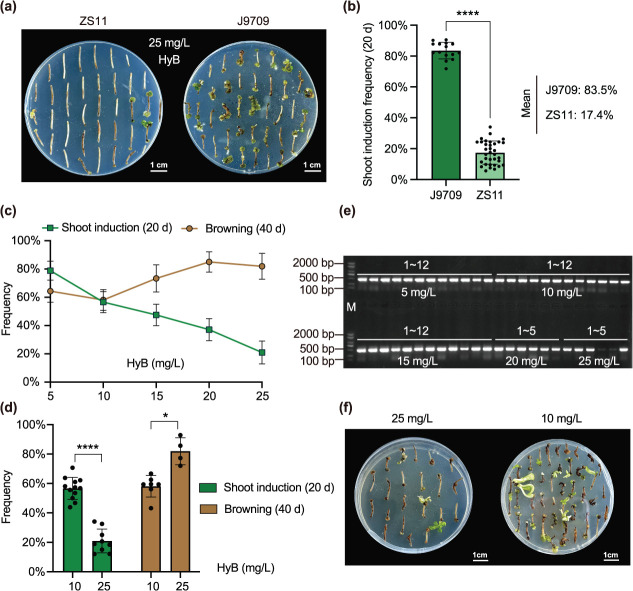
Improvement of regeneration efficiency in ZS11 hypocotyl explants. (a) Phenotype of hypocotyl explants after 20-day shoot induction at 25 mg/L HyB. (b) Shoot induction frequency in ZS11 and J9709. Data are represented as mean ± standard deviation (SD). Asterisks indicate significant differences (^*^P < 0.05, ^**^P < 0.01, Student’s *t-*test). (c) Shoot induction and browning frequency in ZS11 at different HyB concentration. (d) Determination of optimal HyB concentration for ZS11 regeneration. (e) Evaluation of transformation efficiency at different HyB concentrations. The presence of a single bright band is interpreted as a transgenic plant. (f) Phenotype of ZS11 hypocotyl explants after 40-day shoot induction at indicated HyB concentration.

To generate ZS11 transgenic plants, we employed the *Agrobacterium*-mediated hypocotyl transformation and *in vitro* regeneration (Fig. S2). However, ZS11 showed poor regeneration efficiency under the previously established protocol [17–19], with a low shoot induction frequency of 17.4% (Fig. 2a and b). Moreover, regenerated plantlets suffered from browning, hyperhydricity, and root rot, leading to low survival rates. These results indicate that the general protocol for rapeseed regeneration is not optimal for ZS11.

We hypothesized that high antibiotic selection pressure critically suppresses plant regeneration. To test this, we applied a gradient of hygromycin B (HyB) concentrations in ZS11 to determine the optimal level that balances effective selection with regeneration efficiency. As shown in Fig. 2c, shoot induction frequency increased as HyB concentration decreased from 25 to 5 mg/L, while the lowest browning frequency was observed at 10 mg/L. To this end, we selected 10 mg/L as the optimal concentration, as it significantly increased the shoot induction frequency to 56.7%, and reduced the browning frequency from 82.0% to 58.1% (Fig. 2d). Moreover, it ensured a 100% positive rate, with all the 12 randomly selected regenerated plantlets testing transgene-positive (Fig. 2e). Although 5 mg/L yielded the highest shoot induction frequency and also achieved 100% positive rate, the healthier regeneration observed at 10 mg/L supported a more reliable and sustainable protocol (Fig. 2f). Furthermore, we discovered that root robustness could be greatly enhanced by replacing the MS medium with B5 medium, which was reported by a previous study [20]. Taken together, the optimized protocol works well for ZS11 regeneration and will be used in our current and future study to enlarge the knockout collection.

### Editing evaluation of ZS11 CRISPR mutant collection

**Figure 3:**
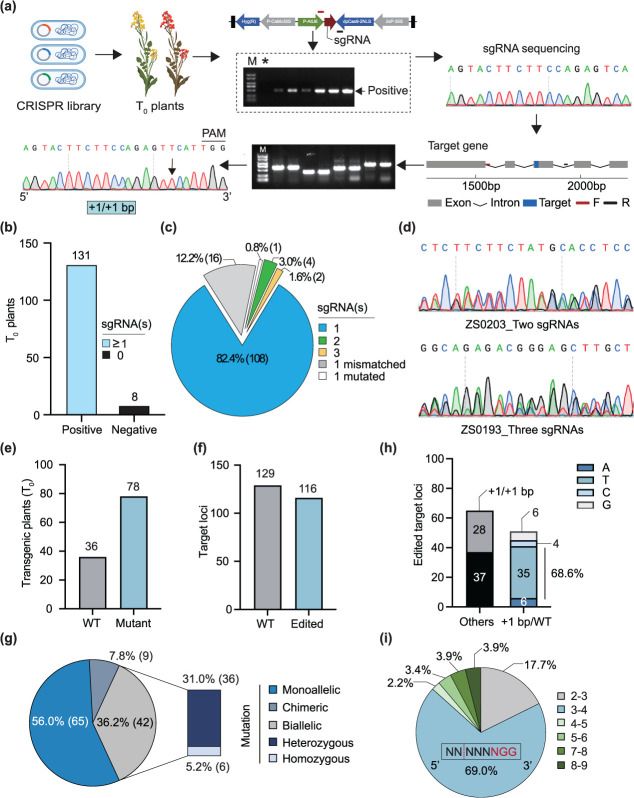
Mutagenesis frequency and editing types in ZS11 mutant collection. (a) Workflow for sgRNA identification and gene editing interrogation. (b) Positive rate in 139 T_0_ plants. (c) sgRNA distribution in 131 transgenic plants. (d) Representative sequencing chromatograms of sgRNAs. (e) Mutagenesis frequency in 114 transgenic plants. (f) Editing frequency in 245 target loci across the 78 mutants. (g) Editing type in 116 edited loci. (h) Repair outcome in 116 edited loci. (i) The position where Cas9-induced DNA double-stranded breaks occur.

Using the optimized protocol, we generated a total of 326 independent T_0_ lines over multiple transformation–regeneration rounds (Fig. 3a). To test the efficiency of *Agrobacterium*-mediated transformation, a pair of specific primers (sgRNA-F/R) was designed to identify sgRNAs in the first batch of 139 T_0_ plants; molecular characterization of the remaining T_0_ lines is currently ongoing. Polymerase chain reaction (PCR) amplification results showed that 131 plants harbored at least one sgRNA (Fig. 3b), indicating a high positive rate of 94.2% (131/139). Sequencing results (Table S4) then showed that 125 plants contained one sgRNA, four plants contained two distinct sgRNAs, while two plants contained three sgRNAs (Fig. 3c and d). These results suggested that most (95.4%) of the transgenic rapeseed plants harbored a ‘single sgRNA’, which is consistent with the previous report [12].

Next, using the 20-nt sgRNA as a ‘proxy’, we decoded the target genes in 114 transgenic plants, excluding 17 plants that harbored mismatched or mutated sgRNAs (Table S4). Editing interrogation (Table S5) revealed that 78 plants contained edited alleles (Fig. 3e), indicating a mutagenesis frequency of 68.4% (78/114), which is higher than that of our previous knockout collection (55.8%). Accordingly, a total of 116 target loci were edited across the 78 mutants (Fig. 3f), comprising 65 monoallelic, 42 biallelic, and 9 chimeric mutations (Fig. 3g). Further analysis revealed that most edited loci (68.1%) were single-base insertions (+1 bp) (Fig. 3h), which is consistent with previous reports [10, 21, 22]. Interestingly, it was discovered in this study that the ‘insertions’ dominated in the form of ‘T-A’, with a high frequency of 68.6% (Fig. 3h), suggesting a potential nucleotide insertion bias. Notably, in-frame deletions were also detected, as exemplified by mutant ZS0176-T_0_, which carried −3 and −6 bp deletions in the *BnATERS.A06* gene (Fig. S5f–i). In addition, most (69.0%) of the cleavage sites by Cas9 were 3 bp upstream of the protospacer-adjacent motif (PAM, 5′-NGG-3′) sequences (Fig. 3i).

To further test the off-target effects of CRISPR-Cas9 system, we predicted the genome-wide off-target sites for five randomly selected T_0_ plants using the Cas-OFFinder tool [23], and finally identified 10 potential off-target sites. None of these sites were edited (Table S6), indicating a low off-target effect of the CRISPR-Cas9 system. Moreover, the edited loci could be stably inherited into the successive generations (Fig. S3).

### Phenotypic screening of the rapeseed knockout collection

**Figure 4:**
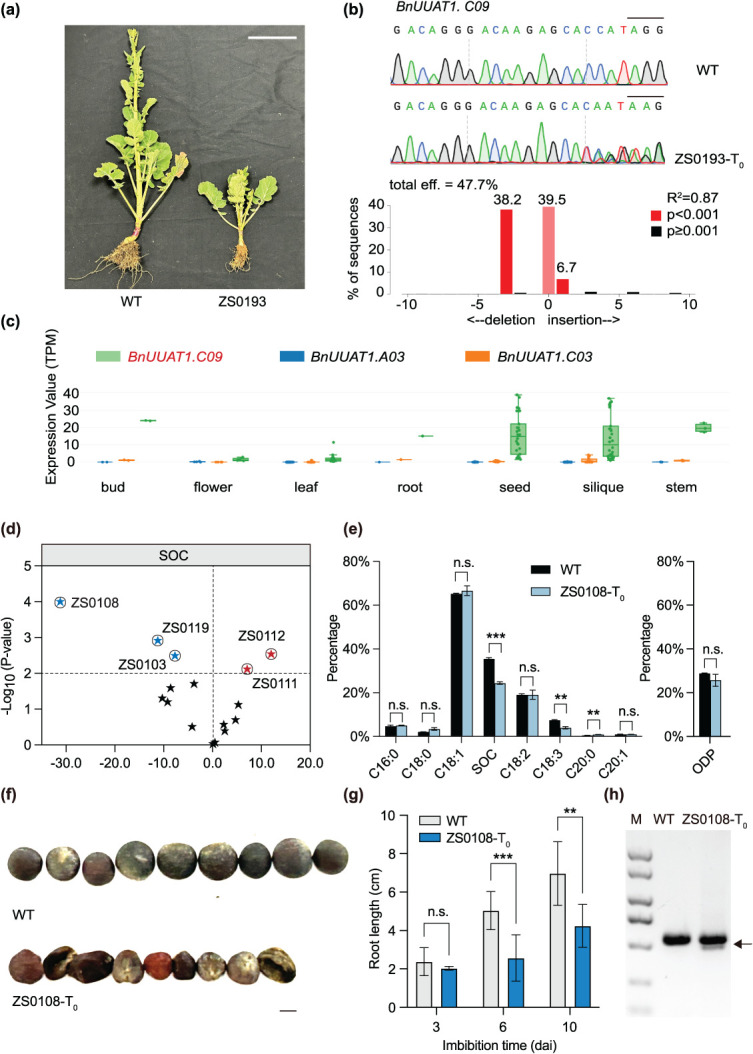
Characterization of two mutants with dwarfism or altered SOC. (a) Phenotype of ZS0193-T_0_ showing the dwarfism. Images of the WT and mutant plants were captured at the same developmental stage. Scale bar, 10 cm. (b) Genotyping of ZS0193-T_0_. Sanger sequencing (upper panel) and TIDE analysis (lower panel) revealed a chimeric mutation. (c) Expression analysis of *BnUUAT1* genes in different tissues of WT rapeseed. (d) GC analysis of SOC in16 randomly selected T_0_ plants. GC, gas chromatography. (e) Fatty acid profile of seeds in ZS0108-T_0_. Asterisks indicate significant differences (^**^P < 0.01, ^***^P < 0.001, Student’s *t-*test); n.s., not significant; ODP, oleic desaturation proportion. (f) Seed morphology of WT and ZS0108-T_0_. Scale bar, 1 cm. (g) Root length of seed germination in ZS0108-T_0_. dai, day after imbibition. (h) PCR amplification of the target locus in ZS0108-T_0_. The arrow shows the edited allele with a 70-bp deletion.

Under field conditions in Wuhan, Hubei Province, China (the rapeseed transgenic base of Huazhong Agricultural University), diverse phenotypic alterations were observed in the T_0_ population (Fig. S4), providing valuable resources for dissecting the genetic basis of key agronomic traits. For example, the ZS0193-T_0_ plant displayed a dwarf and compact architecture (Fig. 4a). Further genotyping revealed that one *BnUUAT1* (*UDP-Uronic Acid Transporter*) homoeolog (*BnUUAT1.C09*) was knocked out in the ZS0193-T_0_ plant (Fig. 4b). In ZS11 wild-type (WT) plants, expression analysis showed that *BnUUAT1.C09* is the predominantly expressed homoeolog in stems, with a significantly higher transcript level than its other two homoeologs (Fig. 4c). This implies that *BnUUAT1.C09* likely plays the primary role among its homoeologs in stem development. Thus, its single knockout could be sufficient to cause a strong dwarf phenotype, uncovering a previously unknown crucial role for this specific homoeolog. This representative example showcased that our knockout collection could facilitate the discovery of novel gene functions in rapeseed. Nevertheless, the findings require further consolidation in advanced generations.

**Figure 5:**
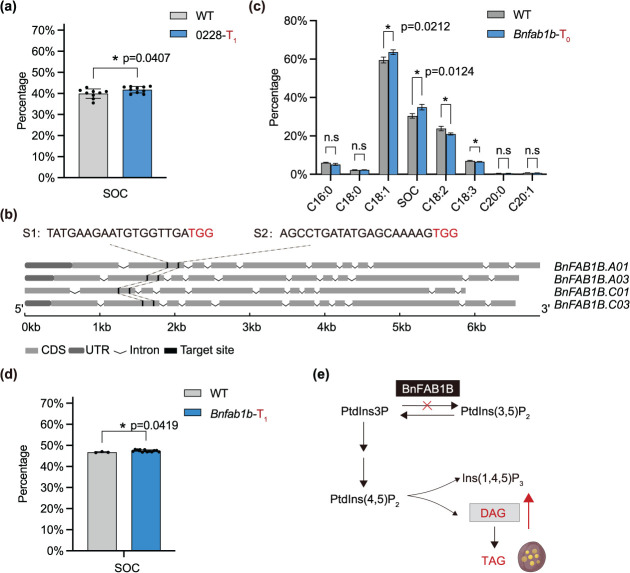
Functional validation of genes in seed metabolism. (a) NIRS analysis of SOC in 0228-T_1_ plants with a knockout in one *BnFAB1B* homoeolog. NIRS, near-infrared spectrometer. Data are represented as mean ± SD. Asterisks indicate significant differences (^*^P < 0.05, Student’s *t-*test). (b) Gene structure of the four *BnFAB1B* homoeologs. (c, d) GC analysis of SOC in *Bnfab1b*-T_0_ (c) and *Bnfab1b*-T_1_ (d) plants with knockouts in all four *BnFAB1B* homoeologs. GC, gas chromatography. (e) Proposed working model for *BnFAB1B*. DAG accumulation is promoted by knocking out the *BnFAB1B* gene. TAG, triacylglycerol.

To further screen for mutants with altered SOC or fatty acid composition, T_1_ seeds harvested from the T_0_ independent plants were analyzed by gas chromatography (Table S7). Several plants with significant changes in SOC (Fig. 4d) or fatty acid composition (Fig. S5a) were identified. For example, the SOC in ZS0108-T_0_ was significantly decreased by 31.2% (Fig. 4e), accompanying with abnormalities in seed morphology and root length (Fig. 4f, g; Table S8); the SOC and oleic acid (C18:1) content in ZS0112-T_0_ were increased by 12.0% and 3.2%, respectively (Fig. S5b), whereas the C18:1 content in ZS0136-T_0_ was decreased by 9.3% (Fig. S5c). Genotyping results showed that all these lines contained edited alleles (Fig. 4h; Fig. S5d, e). Taking ZS0108-T_0_ as an example, a small-fragment deletion was detected in one C09 homoeolog of the *BnER-ANT1* (*endoplasmic reticulum-adenine nucleotide transporter 1*) gene (Fig. 4h), indicating that loss-of-function of *BnER-ANT1.C09* might disrupt the seed oil accumulation. Notably, these findings are consistent with reports in *Arabidopsis*, where loss-of-function of *AtER-ANT1* leads to abnormal seed development and reduced oil content [24], highlighting the reliability of our knockout collection for functional validation of the previously reported genes. Likewise, these results need to be confirmed in subsequent generations.

### Functional validation of the *BnFAB1B* and *BnEDA32* genes

**Figure 6:**
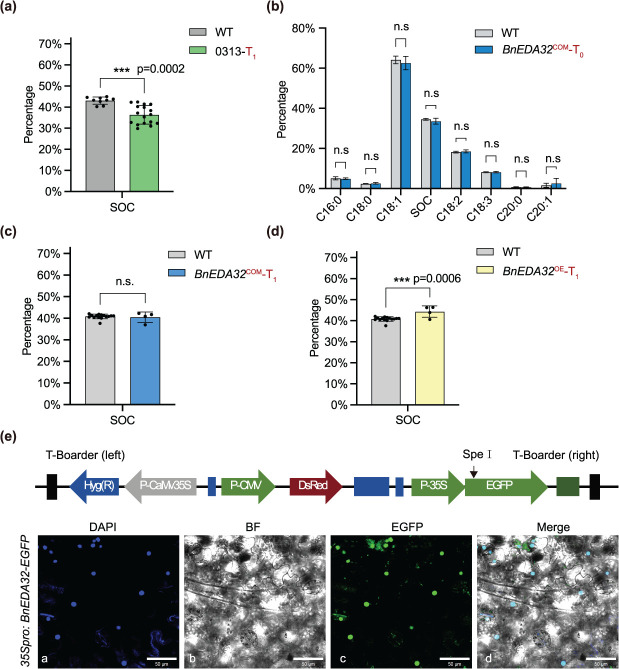
Functional validation of genes in seed metabolism. (a) NIRS analysis of SOC in 0313-T_1_ plants with knockouts in all four *BnEDA32* homoeologs. NIRS, near-infrared spectrometer. Data are represented as mean ± SD. Asterisks indicate significant differences (^***^P < 0.001, Student’s *t-*test). (b) GC analysis of SOC in *BnEDA32*^COM^-T_0_ plants. GC, gas chromatography. (c, d) NIRS analysis of SOC in *BnEDA32*^COM^-T_1_ (c) and *BnEDA32*^OE^-T_1_ (d) plants. (e) Subcellular localization of BnEDA32-EGFP in tobacco cells. BF, bright field; Scale bar, 50 μm.

From our previously established rapeseed knockout collection [13], we identified two candidate genes associated with SOC; knockout of one *BnFAB1B* homoeolog in line 0228-T_0_ significantly increased SOC by 26.8%, whereas knockout of all four *BnEDA32* homoeologs in line 0313-T_0_ reduced SOC by 36.7%. In this study, we confirmed that SOC in 0228-T_1_ remained significantly elevated by 4.8% (Fig. 5a), and SOC in 0313-T_1_ was reduced by 15.6% (Fig. 6a), consistent with the T_0_ observations. Based on the stable phenotypic alterations in the T_0_–T_1_ generations, we prioritized *BnFAB1B* and *BnEDA32* for in-depth analysis.

While the single-homoeolog knockout of *BnFAB1B* increased SOC, we sought to evaluate the combined effect of all *BnFAB1B* homoeologs (Table S9) on seed oil metabolism. Using a dual-sgRNA CRISPR-Cas9 system (Fig. 5b), we generated six independent *Bnfab1b* mutant lines with four *BnFAB1B* homoeologs thoroughly knocked out, and no off-target editing was observed (Fig. S6a–i). As shown in Fig. 5d, SOC in *Bnfab1b-*T_1_ was significantly increased by 1.4%, consistent with the T_0_ observations (Fig. 5c), indicating that knocking out all *BnFAB1B* homoeologs could also promote SOC. Further mechanisms should be investigated to elucidate the precise regulatory pathways through which *BnFAB1B* influences the seed oil metabolism.

Next, to validate that SOC reduction was directly attributable to the *BnEDA32* knockout, we first generated *BnEDA32* complementary lines (Fig. S7a and b). As expected, introduction of a functional *BnEDA32* allele restored SOC to WT levels (Fig. 6b and c), whereas its overexpressing increased the SOC significantly by 8.4% (Fig. 6d). Collectively, these data indicated that BnEDA32 might be a positive regulator of oil accumulation in rapeseed seeds. Subcellular experiments further revealed that BnEDA32 is localized in the nucleus (Fig. 6e), and is predicted to interact with four potential proteins (Fig. S7c) that are associated with DNA repair, redox reactions, and photosynthesis (Fig. S7d). These results suggested a complex regulatory role of BnEDA32 involved in seed lipid metabolism, which needs further investigation.

## Discussion

### The universality of the pooled CRISPR library across different rapeseed varieties

CRISPR-Cas9 system enables a targeted, precise method for introducing genome-scale mutations, providing a platform for high-throughput screening [25, 26]. In our previous work [13], we generated a pooled CRISPR library for rapeseed based on the Darmor-*bzh* reference genome, which comprises 18 414 sgRNAs targeting 10 480 genes, and a J9709 CRISPR mutant collection consisting of 1104 T_0_ lines was then constructed. In this study, we selected the commercial cultivar ZS11 as the new recipient, which outperformed J9709 and other rapeseed varieties [27]. Although sequence variations exist in different rapeseed cultivars [28–30], we show that 15 426 sgRNAs (83.8%) out of the 18 414 guides can be perfectly mapped to the ZS11 genome, targeting 10 078 genes, which is comparable to the number based on the Darmor-*bzh* genome. These numbers can be even larger when we consider those sgRNAs with at most 2-bp mismatch at the 5′ site as effective, as they are also able to induce targeted editing [31]. In this regard, our pooled CRISPR library is universally applicable as a high-throughput mutagenesis tool in different rapeseed varieties and has been distributed to many laboratories. To further enlarge the scale of the mutant library and enhance the gene coverage, we are currently collaborating with other laboratories to increase the number of transgenic T_0_ plants, thereby improving the output of editing events.

### The impact of HyB concentration on selection and regeneration

*Agrobacterium*-mediated transformation and subsequent explant-based regeneration are widely used to establish transgenic plants [32]. Hygromycin B (HyB), an aminoglycoside antibiotic, is commonly utilized as a selection compound to screen for transgene-positive plants [33]. Previous studies revealed that explants from different genotypes show different sensitivity to the HyB concentration [34], resulting in distinct optimal HyB concentrations within even different varieties of the same species. For instance, rapeseed cultivars Huyou-19 and May Slow have an optimal HyB concentration of 6 mg/L, and Suzhou Qing thrives at 9 mg/L [35].

HyB has a significant toxic effect on plant growth and development, thus reducing the efficiency of callus induction and shoot regeneration [33]. In this study, we discovered that the shoot induction frequency of ZS11 hypocotyl explants was significantly lower than that of J9709 at the HyB concentration of 25 mg/L (Fig. S2a and b). To address this issue, we decreased the HyB concentration from 25 to 10 mg/L and found a dramatic increase in shoot induction frequency (Fig. S2c–f). Additionally, the optimized HyB concentration displayed effective selection on the transgene-positive plants while minimizing the toxicity on the hypocotyl explants, with a high transformation efficiency of 94.2% (Fig. 3b). The fine-tuning of the HyB concentration in this study highlights the importance of balancing the defense and growth in the process of generating transgenic plants.

### The possible mechanism of BnFAB1B and BnEDA32 affecting the SOC

FAB1B functions as phosphatidylinositol (PtdIns) 3,5-kinase, catalyzing the substrate PtdIns 3-phosphate (PtdIns3*P*) to the product PtdIns 3,5-bisphosphate [PtdIns(3,5)*P*2] [36]. Previous studies revealed that PtdIns(4,5)*P*2, an isomer of PtdIns(3,5)*P*2, can be hydrolyzed by phospholipase C (PI-PLC), producing inositol 1,4,5-trisphosphate (IP3) and diacylglycerol (DAG) [37]. Notably, DAG is the immediate precursor to triacylglycerol (TAG), which is the major storage form of seed oil [38]. Thus, we speculated that the loss-of-function in the *BnFAB1B* gene could promote DAG accumulation through the PtdIns metabolism pathway, thereby promoting TAG accumulation in seeds (Fig. 5e). While the consistent knockout phenotype confirms *BnFAB1B* as a negative regulator of oil accumulation, deeper mechanistic insights, including those from complementation and overexpression studies in the polyploid rapeseed background, will be the focus of future work. In addition, loss of function in *AtFAB1B* was shown to impair endomembrane homeostasis, conferring pleiotropic developmental abnormalities in *Arabidopsis* [39]. These results indicated a potential functional divergence of *FAB1B* across different species.

The BnEDA32 protein was predicted to harbor the NYN (Nedd4-BP1, YacP nucleases, and Nedd4-BP1) domain. Previous studies elucidated that the disruption of the *AtEDA32* gene would result in unfused polar nuclei in *Arabidopsis* [40]. Thus, we speculated that knocking out the *BnEDA32* gene could influence seed oil biosynthesis by disrupting critical cellular function or metabolic regulation, thereby resulting in a reduction of TAG levels (Fig. S7d). In future studies, we will integrate multi-omics data, such as transcriptome and proteome, to reveal the specific regulatory mechanisms of *BnFAB1B* and *BnEDA32* genes in seed lipid metabolism, providing deeper insights into breeding novel varieties associated with both genes.

In this study, we focused on functional validation of *BnFAB1B* and BnEDA32 because their knockout lines exhibited stable, reproducible effects on seed oil content across generations (T_0_–T_1_), providing a solid basis for mechanistic investigation. Meanwhile, the promising candidate genes newly identified from the mutant library, such as *BnUUAT1.C09* and *BnER-ANT1.C09*, require phenotypic consolidation in advanced generations, which constitutes an important direction for our future work.

## Conclusion

This study has enlarged the rapeseed mutant collection with different varieties by employing our pooled CRISPR library as a high-throughput mutagenesis tool in the elite cultivar ZS11. Through an optimized regeneration protocol, we have generated a knockout collection in ZS11 with a wide range of phenotypic variations. Importantly, the scale-expansion of the mutant library has also facilitated the identification of novel genetic modifications, and we further demonstrated that the knockout of the *BnFAB1B* gene can lead to a significant increase in SOC, underscoring the potential agricultural applications. Together, our study provides valuable genetic resources for rapeseed functional genomics and elite germplasm breeding.

## Materials and methods

### Plant materials and growth conditions

All WT and transgenic rapeseed plants were cultivated in the field under natural conditions in Wuhan, Hubei Province (semiwinter rape-producing area, 114.44°E, 30.53°N). Tobacco plants (*Nicotiana benthamiana*) used for subcellular localization were grown in a greenhouse at 25°C under a light/dark cycle of 16/8 h.

### CRISPR library construction and sgRNA mapping

The pooled CRISPR library used in this study was constructed previously [13]. The target genes were selected based on a defined biological rationale: *A. thaliana* orthologs associated with reproductive development were combined with *B. napus* genes showing high expression during seed development. From this curated set, 18 414 sgRNAs were designed, synthesized as pooled oligonucleotides, annealed into double-stranded fragments, and ligated in bulk into the linearized CRISPR-Cas9 vector DP-05. This preconstructed library was transformed into *Agrobacterium tumefaciens* for plant transformation. In this study, all 18 414 sgRNA sequences were computationally mapped to the ZS11 genome using a custom Python script, and 15 426 sgRNAs aligned perfectly (zero mismatches) to ZS11 (Table S2).

### Vector design and construction

The CRISPR-Cas9 target site was designed using the online tool (http://crispr.hzau.edu.cn/CRISPR2/) [41], and the dual-sgRNA vector for knocking out the four *BnFAB1B* homoeologs was constructed using DP-05 vector as the backbone. The coding sequence (CDS) of *BnEDA32* was cloned into the pCAMBIA1303-FLAG plasmid to construct the complementary vector, and the CDS of *BnEDA32* excluding stop codon was cloned into the pCAMBIA1303-EGFP plasmid to construct the subcellular-localization vector. All of the plasmids were separately electroporated into the *A. tumefaciens* strain GV3101. Primers are listed in Table S10.

### Rapeseed transformation and regeneration

The rapeseed seeds were surface-sterilized with 70% ethanol (1 min) and 50% bleach (3 min), then germinated on half-strength MS medium in the dark at 25°C for 1 week to elongate hypocotyls. Hypocotyls were cut into 0.5-cm segments and immersed in *Agrobacterium* suspension for 10 min, cocultivated in the dark at 25°C for 2 days, and subsequently transferred to callus-, shoot- and root-induction media at 25°C under 16 h light/8 h dark (3300 lx). Regenerated plantlets were grown in a greenhouse at 24°C (16 h light/8 h dark, 2500 lx) for 3 weeks before being transplanted to the field. The entire transformation process roughly takes 5 months.

### sgRNA identification and gene editing interrogation

Genomic DNA was extracted from leaf tissue of each independent T_0_ line with the NuClean Plant Genomic DNA Kit (CW0531S). Transgene-positive plants were identified by amplifying the sgRNA using a pair of primers (Table S10); amplicons were visualized on 1% agarose gels, and Sanger-sequenced. A custom Python script (Cartesian-product algorithm) was developed to deconvolve superimposed sequencing chromatograms of sgRNA(s) and identify the specific 20-nt sgRNA(s) present in each plant, which were then mapped to the target genes. For each target site, locus-specific primers were used to amplify genomic fragments from every transgenic plant. WT PCR products served as negative controls. TIDE [42] or ICE [43] software was utilized to analyze the editing outcome of target loci. Primers are listed in Table S10.

### Off-target analysis of the CRISPR-Cas9 system

All potential off-target sites were predicted leveraging the stand-alone Cas-OFFinder software [23], and several top sites were randomly selected for editing interrogation. The primers used for amplifying the off-target sites are listed in Table S6 and Table S10.

### Phenotyping of the seed-related traits

SOC and fatty acid composition were measured with a NIRS Systems 5000 (Foss, Denmark) [44, 45]. For precise fatty acid profiling, analysis was conducted on an Agilent GC 6850 system following the established protocol [46]. For gas chromatography (GC) analysis, ~20 mg of dry, mature seed was ground in 3 mL methanol, supplemented with 0.8 mL methylbenzene and 0.4 mL heptadecanoic acid (C17:0) internal standard (2 mg/ml in toluene), followed by incubation at 98°C for 1 h. After cooling, 3.6 mL water and 2 mL hexane were added, vortexed, and the hexane layer containing fatty acid methyl esters was recovered for GC injection.

To measure root length of seed germination, mature seeds were placed in moistened germination pouches (Phytotc) and incubated at 25°C in darkness. Root length was then measured at 3, 6, and 10 days after imbibition (dai).

### Subcellular localization

*Agrobacterium* GV3101 strain containing the subcellular-localization vector was injected into 5-week-old tobacco leaves. DAPI, a nuclear staining dye, was used to visualize the nuclei of tobacco cells [47, 48]. The enhanced green fluorescent protein (EGFP) signal and DAPI signal were observed under 488 nm and 405 nm, respectively, using the confocal laser scanning biological microscope (FV 1000).

## Supplementary Material

Web_Material_uhag087

## Data Availability

All relevant data are available within this article and supplemental information. Gene and protein sequences are publicly accessible in TAIR (https://www.arabidopsis.org/) [49] and the BnIR databases [50]. The expression data of ZS11 genes used in this study were downloaded from the BnIR database (http://yanglab.hzau.edu.cn/BnIR) [50]. The Python script [13] used for parsing the Sanger reads of the sgRNAs has been deposited at the website of https://github.com/Jianjie-He/Deconvolve_sgRNA. The CRISPR sgRNA library and the mutant seeds generated in this study are available for academic research upon request under a Material Transfer Agreement (MTA) from the corresponding authors.
