# Supplementary material for: A small-scale CRISPR mutant library in rapeseed of commercial cultivar Zhongshuang 11
Source: Hortic Res. 2026 Mar 5;13(7):uhag087. doi: 10.1093/hr/uhag087 (PMC13291919; doi:10.1093/hr/uhag087)
Supplement: Web_Material_uhag087 [file Web_Material_uhag087.zip › Supplementary Figures.pdf]

Supplementary Figures

Fig. S1 Characteristics of the sgRNA and target genes.

Fig. S2 The process of generating ZS11 transgenic plants.

Fig. S3 Evaluation of mutation heritability.

Fig. S4 Field phenotypic variations observed in the T_0 population.

Fig. S5 Phenotyping and genotyping analysis of the T_0 plants.

Fig. S6 Functional validation of *BnFAB1B* gene in seed metabolism.

Fig. S7 Functional validation of *BnEDA32* gene in seed metabolism.

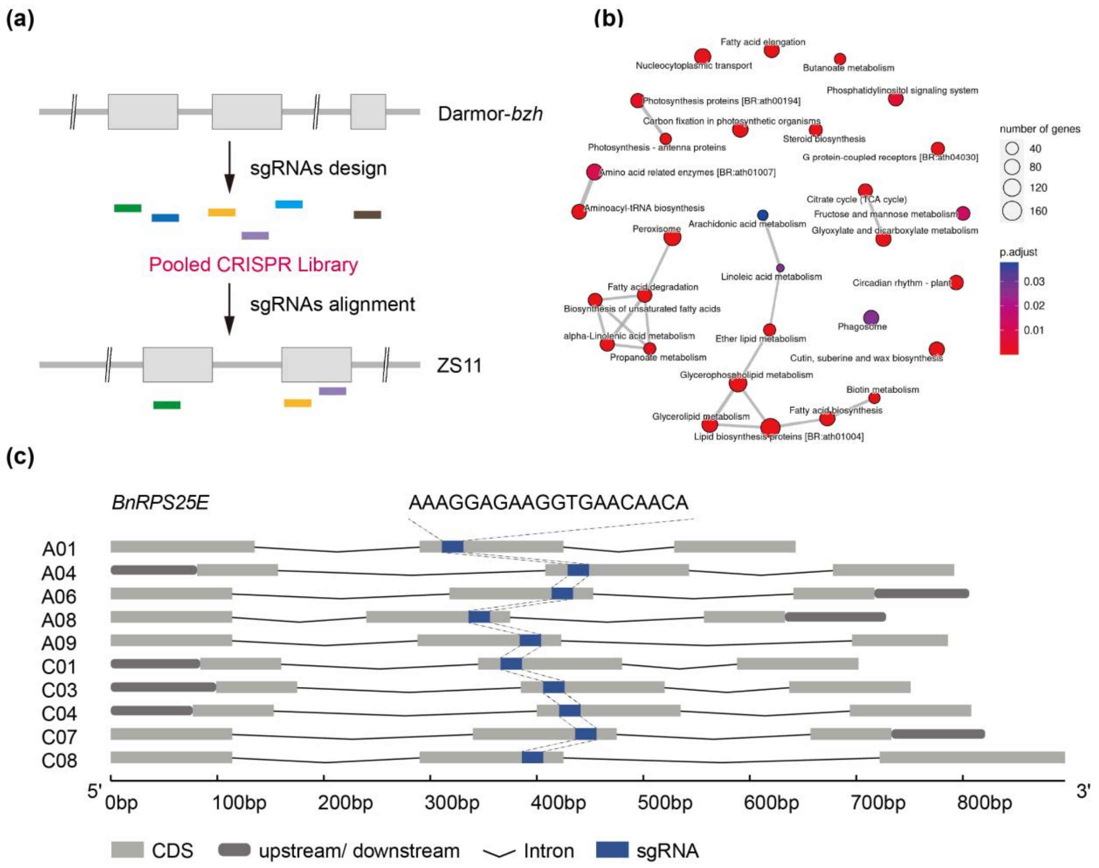

Fig. S1 Characteristics of sgRNA and target genes.

(a) Workflow for mapping the pooled CRISPR library to ZS11 genome. Colored boxes represent distinct sgRNAs; Grey boxes represent exons within the genome.

(b) KEGG enrichment analysis of target genes. KEGG, kyoto encyclopedia of genes and genomes.

(c) Schematic diagram illustrating how one sgRNA can simultaneously target all ten *BnRPS25E* homoeologs. CDS, coding sequence.

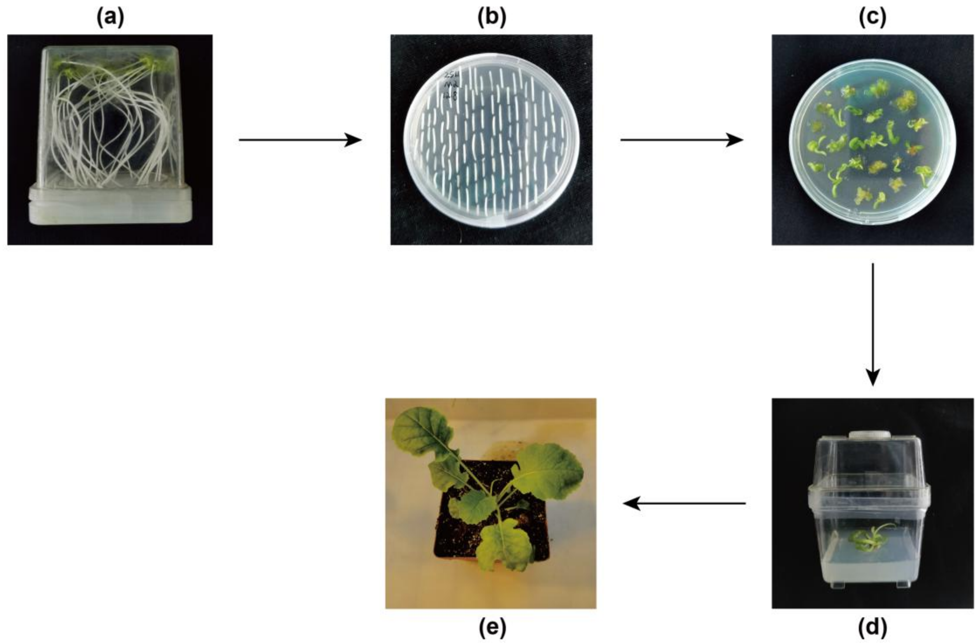

Fig. S2 The process of generating ZS11 transgenic plants. Preparation of the hypocotyl explants **(a)**, *Agrobacterium*-mediated genetic transformation and calluses induction **(b)**, shoot induction **(c),** root induction **(d)**, and transferring the regenerated plants to soil **(e)**.

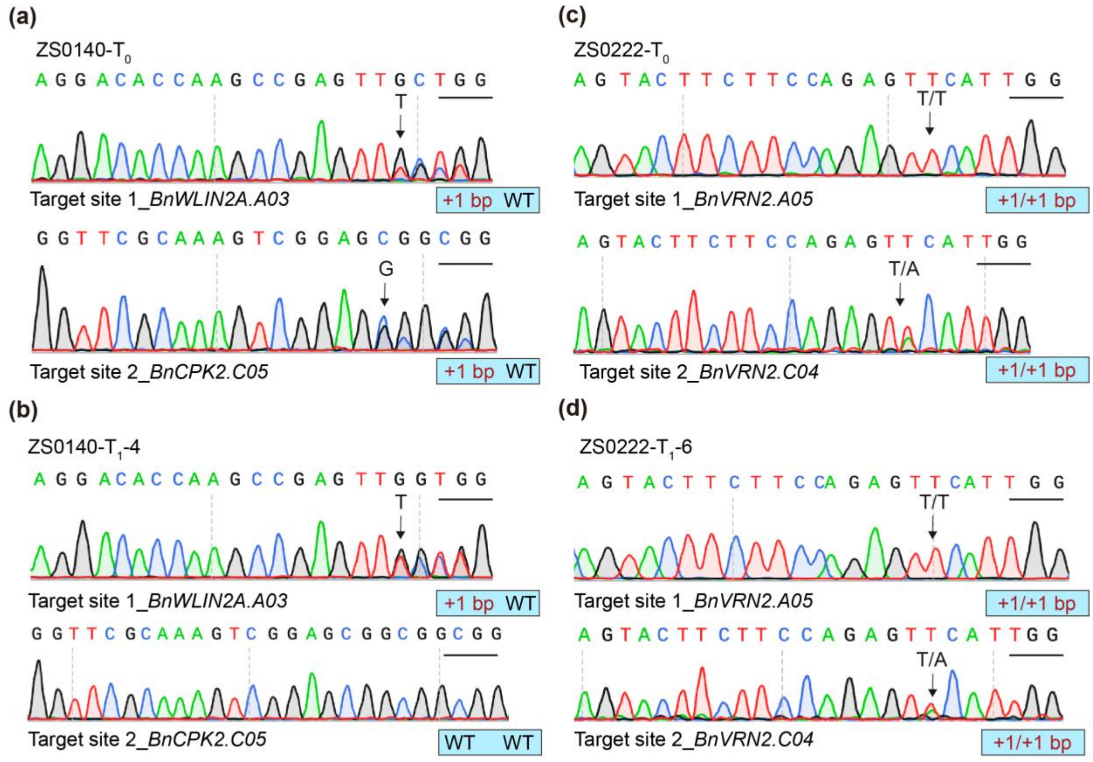

Fig. S3 Evaluation of mutation heritability. Editing interrogation of target loci in ZS0140-T_0 **(a)**, ZS0140-T_1 **(b)**, ZS0222-T_0 **(c)** and ZS0222-T_1 **(d)**.

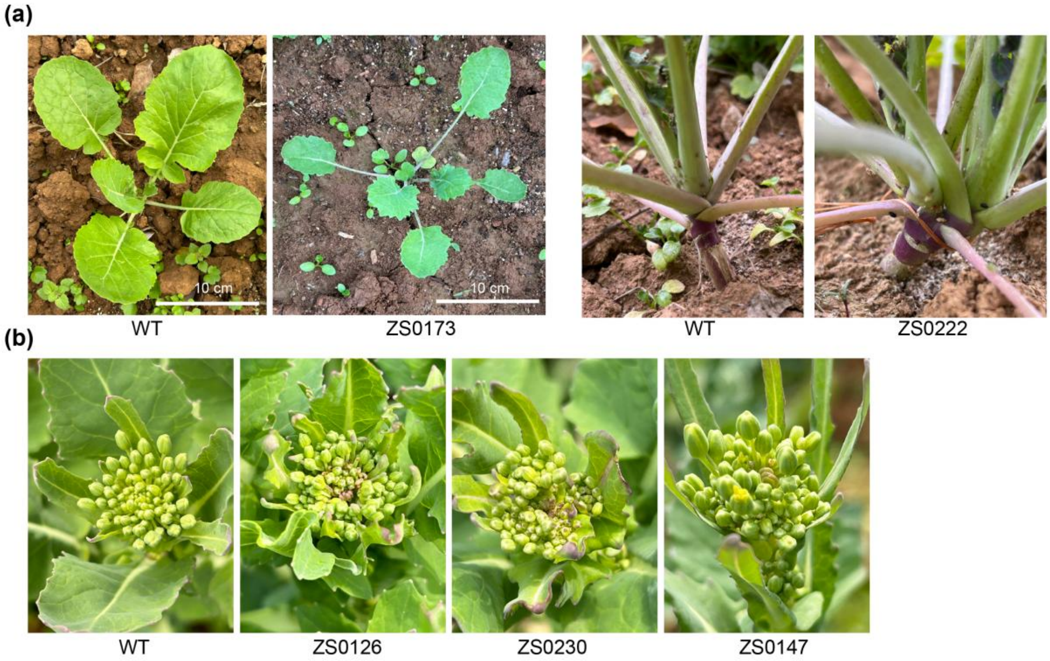

Fig. S4 Field phenotypic variations observed in the T_0 population.

(a) The deeper green and multi-stem phenotypes of lines ZS0173 and ZS0222 during the vegetative stages.

(b) The abnormal inflorescence phenotype of lines ZS0126, ZS0230, and ZS0147 during the reproductive stages.

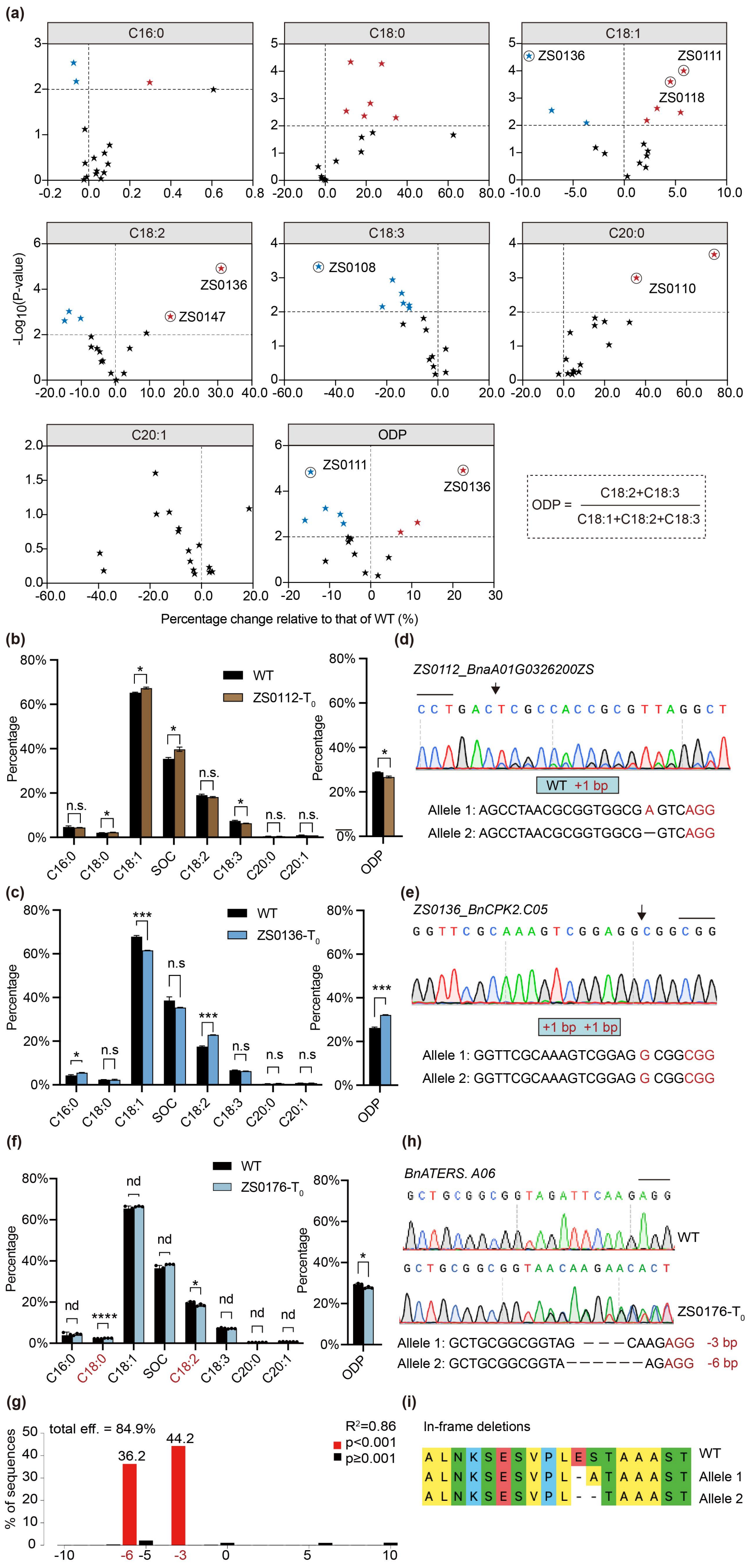

(a)
C16:0
C18:0
C18:1
ZS0136
ZS0111
ZS0118
C18:2
ZS0136
ZS0147
C18:3
ZS0108
C20:0
ZS0110
C20:1
ODP
ZS0111
ZS0136
ODP = C18:2+C18:3 / C18:1+C18:2+C18:3
-Log10(P-value)
Percentage change relative to that of WT (%)
(b)
WT
ZS0112-T0
Percentage
C16:0 C18:0 C18:1 SOC C18:2 C18:3 C20:0 C20:1
ODP
(d)
ZS0112_BnaA01G0326200ZS
CCTGACTCGCCACCGCGTTAGGCT
WT +1 bp
Allele 1: AGCCTAACGCGGTGGCG A GTCAGG
Allele 2: AGCCTAACGCGGTGGCG—GTCAGG
(c)
WT
ZS0136-T0
Percentage
C16:0 C18:0 C18:1 SOC C18:2 C18:3 C20:0 C20:1
ODP
(e)
ZS0136_BnCPK2.C05
GGTTCGCAAAGTCGGAGGCGGCGG
+1 bp +1 bp
Allele 1: GGTTCGCAAAGTCGGAG G CGGCGG
Allele 2: GGTTCGCAAAGTCGGAG G CGGCGG
(f)
WT
ZS0176-T0
Percentage
C16:0 C18:0 C18:1 SOC C18:2 C18:3 C20:0 C20:1
ODP
(h)
BnATERS. A06
GCTGCGGCGGTAGATTCAAGAGG
WT
GCTGCGGCGGTAACAAGAACACT
ZS0176-T0
Allele 1: GCTGCGGCGGTAG – – – CAAGAGG -3 bp
Allele 2: GCTGCGGCGGTA––––––AGAGG -6 bp
(g)
total eff. = 84.9%
R²=0.86
p<0.001
p≥0.001
% of sequences
36.2
44.2
(i)
In-frame deletions
ALNKSESVPLESTAAAST WT
ALNKSESVPL-ATAAAST Allele 1
ALNKSESVPL--TAAAST Allele 2

Fig. S5 Phenotyping and genotyping analysis of the T_0 plants.

(a) Fatty acid profiling of seeds in 16 randomly selected T_0 plants. Each dot represents an independent T_0 line, and the red and blue ones indicate a significant increase and decrease, respectively. ODP, oleic desaturation proportion.

(b-c) Fatty acid profiling of seeds in ZS0112-T_0 **(b)** and ZS0136-T_0 **(c)**. Asterisks indicate significant differences (*P < 0.05, ***P < 0.001, Student's *t* test); n.s., not significant.

(d-e) Editing interrogation of ZS0112-T_0 **(d)**, and ZS0136-T_0 **(e)**. Arrows indicate the cleavage sites, and the black lines indicate PAM.

(f-i) Phenotype-to-genotype analysis of ZS0176-T_0. Black lines indicate PAM.

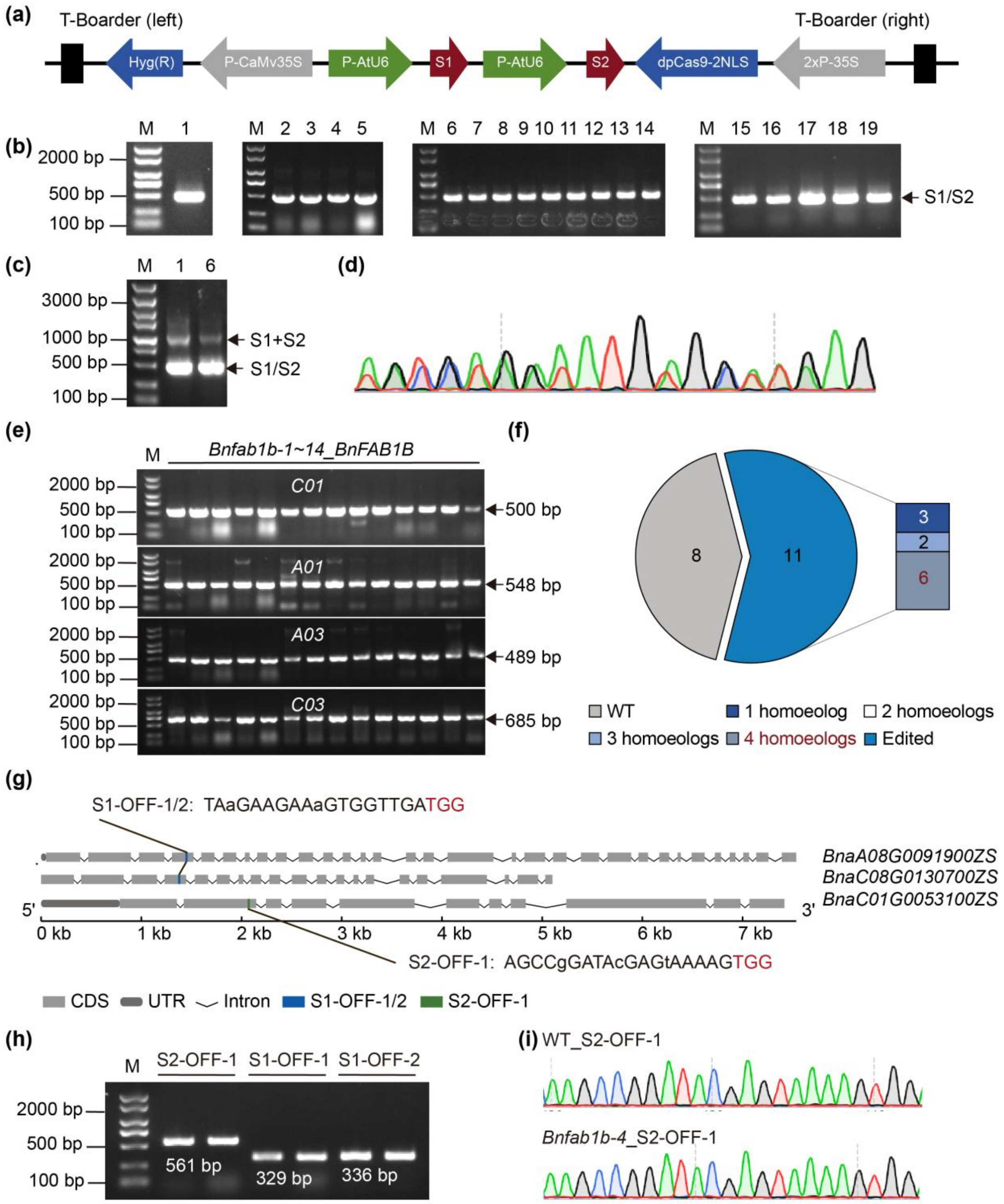

Fig. S6 Functional validation of *BnFAB1B* gene in seed metabolism.

(a-f) Effective editing of four *BnFAB1B* homoeologs using dual sgRNAs. **(a)** Schematic diagram of the dual-sgRNA vector. S1, sgRNA1; S2, sgRNA2. **(b)** sgRNA amplification in T_0 plants. **(c)** Sanger sequencing of sgRNA1 and sgRNA2. **(d)** Amplification of the four target loci in 19 transgenic T_0 plants. **(f)** Summary of the editing outcome of the 19 T_0 plants.

(g-i) Off-target analysis of CRISPR-Cas9 system. **(g)** Schematic diagram of the potential off-target sites and their corresponding genes. The mismatched nucleotides

are marked in lowercase. **(h)** PCR amplification of the selected off-target sites. **(i)** Editing interrogation of the selected off-target sites.

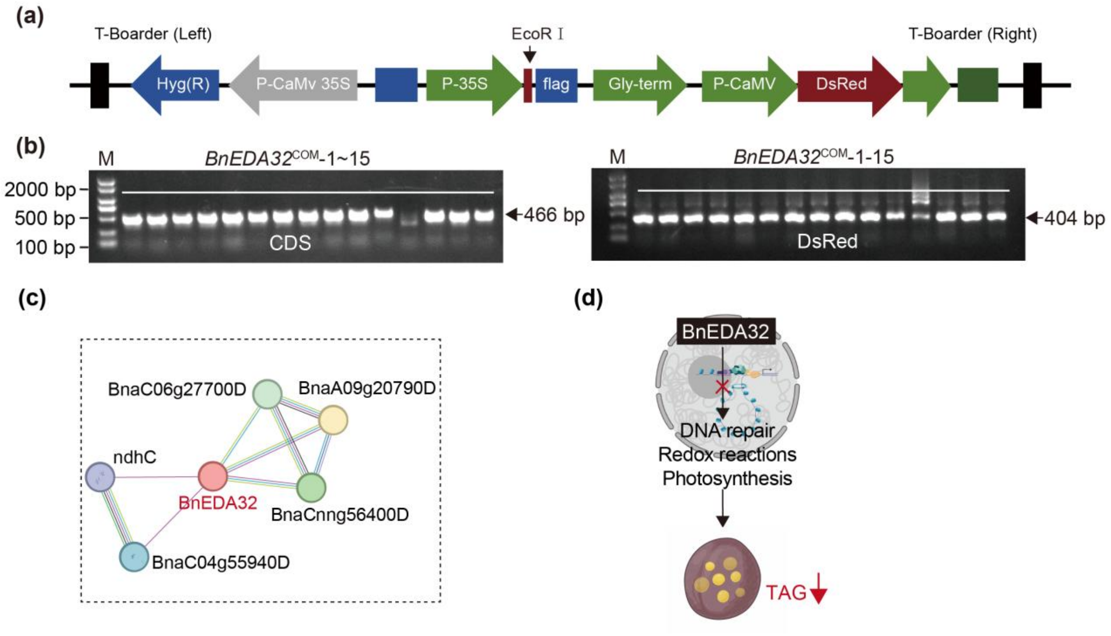

Fig. S7 Functional validation of *BnEDA32* gene in seed metabolism.

(a) Schematic diagram of the vector used for overexpression the *BnEDA32* gene.

(b) Identification of the transgene-positive plants via PCR amplification of the CDS (left) and DsRed (right) sequences.

(c) Protein-protein interaction network of BnEDA32.

(d) Proposed working model for BnEDA32. TAG, triacylglycerol.
